# Hyperlipidemia and Statins Use for the Risk of New Diagnosed Sarcopenia in Patients with Chronic Kidney: A Population-Based Study

**DOI:** 10.3390/ijerph17051494

**Published:** 2020-02-26

**Authors:** Min-Hua Lin, She-Yu Chiu, Pei-Hsuan Chang, Yu-Liang Lai, Pau-Chung Chen, Wen-Chao Ho

**Affiliations:** 1 Department of Nutrition, China Medical University, Taichung 40402, Taiwan; minhua5356@gmail.com (M.-H.L.); koca345678@gmail.com (S.-Y.C.); 2 Department of Dietetics, Yunlin Christian Hospital, Yunlin 64866, Taiwan; 3 Department of Clinical Nutrition, China Medical University Hospital, Taichung 40402, Taiwan; peipei10220312@gmail.com; 4 Department of Public Health, China Medical University, Taichung 40402, Taiwan; 5 Department of Physical Therapy Medicine and rehabilitation, China Medical University, Hsinchu 302, Taiwan; yuliang22@gmail.com; 6 Department of Physical Therapy and School of Medicine, China Medical University, Taichung 40402, Taiwan; 7 Department of Public Health, National Taiwan University, Taipei 10617, Taiwan; pchen@ntu.edu.tw

**Keywords:** chronic kidney disease, statins, sarcopenia

## Abstract

*Background*: Previous research found that statins, in addition to its efficiency in treating hyperlipidemia, may also incur adverse drug reactions, which mainly include myopathies and abnormalities in liver function. *Aim:* This study aims to assess the risk for newly onset sarcopenia among patients with chronic kidney disease using statins. *Material and Method:* In a nationwide retrospective population-based cohort study, 75,637 clinically confirmed cases of chronic kidney disease between 1997 and 2011were selected from the National Health Insurance Research Database of Taiwan. The selection of the chronic kidney disease cohort included a discharge diagnosis with chronic kidney disease or more than 3 outpatient visits with the diagnosis of chronic kidney disease found within 1 year. After consideration of patient exclusions, we finally got a total number of 67,001 cases of chronic kidney disease in the study. The Cox proportional hazards model was used to perform preliminary analysis on the effect of statins usage on the occurrence of newly diagnosed sarcopenia; the Cox proportional hazards model with time-dependent covariates was conducted to take into consideration the individual temporal differences in medication usage, and calculated the hazard ratio (HR) and 95% confidence interval after controlling for gender, age, income, and urbanization. *Results:* Our main findings indicated that patients with chronic kidney disease who use statins seem to effectively prevent patients from occurrences of sarcopenia, high dosage of statins seem to show more significant protective effects, and the results are similar over long-term follow-up. In addition, the risk for newly diagnosed sarcopenia among patients with lipophilic statins treatment was lower than that among patients with hydrophilic statins treatment. *Conclusion:* It seems that patients with chronic kidney disease could receive statin treatment to reduce the occurrence of newly diagnosed sarcopenia. Additionally, a higher dosage of statins could reduce the incidence of newly diagnosed sarcopenia in patients with chronic kidney disease.

## 1. Background

An aging population is becoming an increasingly serious trend around the world. Sarcopenia is a chronic disease that decreases muscle mass and function with increasing age, leading to reduced cardiopulmonary functions, degradation of physiological functions, low quality of life, slow metabolism, risks of falling, dysfunction, death, and increased medical expenses among old people [1,2,3,4]. Souza et al. found that patients with sarcopenia are at a higher risk for chronic kidney disease than the general population, and those with chronic kidney disease are more likely to be further suffering from adverse cardiovascular events [5]. In 2002, the Kidney Disease Outcome Quality Initiative (KDOQI) defined chronic kidney disease as “a kidney injury or eGFR lower than 60 mL/min/1.73 m^2^, which persists for more than 3 months.” Pereira et al. found that the incidence rate for sarcopenia is around 5%–9% in patients in the early stages of chronic kidney disease [6]. Chen et al. found that elderly patients with chronic kidney disease (eGFR < 45 mL/min) are at an increased risk of sarcopenia [7]. In addition, Carrero and Steiber et al. used subjective global assessment to provide evidence that muscle wasting is a predictor of mortality in patients with chronic kidney disease and the severity of muscle wasting is associated with the 5-year survival rate [8,9]. Thus, sarcopenia tends to develop among patients in the later stages of chronic kidney disease and is a significant risk factor. Because of the reduced renal function, patients with chronic kidney disease tend to suffer from several comorbid conditions, including dyslipidemia and related diseases, thus resulting in high risk for cardiovascular diseases. Past literature indicated that moderate- to high-intensity statins should be initiated if patients not receiving dialysis had clinical atherosclerotic cardiovascular disease (ASCVD) such as acute coronary syndromes [10]. For patients with dialysis, guidelines of the 2013 kidney disease: improving global outcomes (KDIGO) and the National Kidney Foundation Kidney Disease Outcomes Quality Initiative (KDOQI) advise that statins treatment should not be administered routinely [11,12], Thus, the probability of being exposed to statins is higher in patients with chronic kidney disease than in the general population. However, recent research indicated that statins may increase patients’ risk for myopathies [13,14,15]. Current literature pertaining to the association between chronic kidney disease (CKD) and prognosis of sarcopenia is sparse and controversial. Moreover, there are six main categories of commonly used statins, with nuances in their mechanisms of action and in their synthesis routes, leading to the difference in pharmacological effects, however, the underlying cause–effect relationship and comorbidities require further investigation.

This study aimed to analyze the medication profile of patients with chronic kidney disease in Taiwan and explore whether statins use is associated with an increased risk for sarcopenia among patients with chronic kidney disease or other consequences. In addition, the study explored the effects of statins dose level and the six main categories of statins on the incidence risk for newly diagnosed sarcopenia to prevent the occurrence of sarcopenia among patients with chronic kidney disease.

## 2. Material and Method

### 2.1. Data Source

The data for the present study were retrieved from the Taiwan National Health Insurance Research Database (NHIRD), which covers 99.9% of the Taiwanese population of approximately 23 million. The NHIRD is derived from the Taiwanese National Health Insurance (NHI) program, which was launched in 1995 to finance health care for all citizens. The data used in this cohort study were obtained from the Longitudinal National Health Insurance Research Database (LHID) 2000. For the LHID2000, about 1,000,000 representative individuals were randomly sampled from the NHI Registry of Beneficiaries in 2000. LHID2000 contains the following records: the ambulatory care expenditures by visits (CD), details of ambulatory care orders (OO), inpatient expenditures by admissions (DD), details of inpatient orders (DO), expenditures for prescriptions dispensed at contracted pharmacies (GD), registry for beneficiaries (ID). These records are renewed every year. It is one of the largest medical databases in the world, and has been used to analyze the epidemiology in Taiwan extensively [16]. This study was approved after a full ethical review by the Institutional Review Board (IRB) of the National Taiwan University Hospital (approval number 20151105), and the IRB waived the need for consent [17]. Those data were accessed anonymously.

### 2.2. Definition of Sarcopenia and Selection of ICD Code

In October 2016, the United States Centers for Disease Control and Prevention (CDC) formally recognized sarcopenia as a disease, encoded as M62.84 in ICD-10-CM. However, the code for sarcopenia is absent in ICD-9-CM, which was used by Taiwan’s NHIRD. Therefore, this study followed the suggestion of clinical physicians in practice and referred to the following regulations on code conversion from ICD-9-CM to ICD-10-CM: ICD-9-CM:728.2 can be converted as ICD-10-CM: M62.5 (https://www.icd10data.com/Convert/728.2), and ICD-9-CM: 728.9 can be converted as ICD-10-CM: M62.9 (https://www.icd10data.com/Convert/728.9). In this study, sarcopenia was clinically diagnosed, based on muscle wasting and atrophy (ICD-9-CM: 728.2), and unspecified disorders of muscle, ligament, and fascia (ICD-9-CM: 728.9). This study tracked patients with chronic kidney disease with newly onset sarcopenia, and determined the diagnosis of disease based on the first three digits of the diagnosis codes in the inpatients expenditures by admissions (DD) records for the first records of sarcopenia (ICD-9-CM: 728.2, 728.9). This study aimed to explore the interaction between the newly onset sarcopenia and other factors. Thus, patients who already showed symptoms of sarcopenia prior to their diagnosis of chronic kidney disease were excluded.

### 2.3. Selection of the Chronic Kidney Disease Cohort and Sarcopenia Cases

This study retrieved data of patients with chronic kidney disease from ambulatory care expenditures by visits (CD) and inpatient expenditures by admissions (DD). Among the 995,347 enrolled patients of the LHID2000 Registry for Beneficiaries of the Taiwan NHIRD, the study reviewed 75,637 clinically confirmed cases of chronic kidney disease between 1997 and 2011. The determination of chronic kidney disease was based on the first three digits of the diagnosis codes in medical records and the International Classification of Disease, Ninth Revision, Clinical Modification (ICD-9-CM) adopted as the selection criteria. Records encoded with ICD-9-CM:250.4,274.1,283.11,403.x1, 404.x2, 404.x3, 440.1, 442.1, 447.3, 572.4, 580-588, 642.1x, 646.2 [18], The selection of the chronic kidney disease cohort included a discharge diagnosis with chronic kidney disease or more than 3 outpatient visits with the diagnosis of chronic kidney disease found within 1 year. We excluded those patients with incomplete records (N = 1020), age smaller than 18 (N = 1946), and those who had a diagnosis of sarcopenia prior to January 1, 1997 (N = 73). There were 72,598 cases of newly diagnosed patients with chronic kidney disease. Further exclusion criteria were as follows: those with a medical record of sarcopenia before the diagnosis of chronic kidney disease (1922 cases), those who experienced onset of sarcopenia within 1 year of statins administration, and those who were lost in follow-up (3675 cases). In this study, sarcopenia was clinically diagnosed, based on muscle wasting and atrophy (ICD-9-CM: 728.2), and unspecified disorders of muscle, ligament, and fascia (ICD-9-CM: 728.9). Patients who already showed symptoms of sarcopenia prior to their diagnosis of chronic kidney disease were excluded (N = 1922). These exclusions finally resulted in a total number of 67,001 cases of chronic kidney disease, which were used in the study. This study aimed to explore the interaction between the newly onset sarcopenia and medication usage of statins. We tracked patients with chronic kidney disease with newly onset sarcopenia, and determined the diagnosis of disease based on the first three digits of the diagnosis codes in the inpatients expenditures by admissions (DD) record.

### 2.4. Assessment of Comorbidities and Confounding Factors

Diagnostic criteria of comorbidities with sarcopenia in the research population were based on having at least two outpatient records in a year or one inpatient record, and diseases that were included were: hypertension (ICD-9-CM: 401.x-405.x; A-code: A260, A269), hyperlipidemia (ICD-9-CM: 272.x; A-code: A189), atrial fibrillation and flutter (ICD-9-CM:427.3,427.31;A-code:A281), coronary artery disease (ICD-9-CM: 410.x-414.x,429.2;A-code:A270, A279), heart failure (ICD-9-CM:428.x;A-code:A289), peripheral artery disease (ICD-9-CM: 250.7, 443.x, 444.2; A-code: A302), diabetes (ICD-9-CM: 250,249; A-code: A181), and liver diseases (ICD-9-CM: 571.2, 571.5-571.7, 572.2-572.5, 572.8, 573.0).

### 2.5. Medication Records and Calculation Unit of the National Health Insurance Research Database

The study’s primary drug for analysis is statins, and the six main categories of statins commonly used clinically were included as follows: hydrophilic: pravastatin, fluvastatin, rosuvastatin, and lipophilic: simvastatin, lovastatin, atorvastatin. Medications used by the study subjects before the confirmed diagnosis of sarcopenia included drugs to control hypertension, hyperlipidemia, hyperglycemia, uric acid, gout, and nonsteroidal anti-inflammatory drugs (NSAIDs). According to the 2014 Joint National Committee (JNC8) guidelines for the management of hypertension in adults, the recommended first-line drugs to treat hypertension include diuretics, beta-adrenoceptor antagonists (beta blockers), angiotensin-converting enzyme inhibitors (ACEi), and angiotensin receptor blocker (ARB) [18].

Major drugs for hyperlipidemia management include non-statin drugs to lower blood cholesterol, such as cholestyramine, colestipol, colextran, niceritrol, nicofuranose, acipimox, probucol, and ezetimibe, and fibric acid derivatives (fibrates), such as bezafibrate, clofibrate, etofibrate, fenofibrate, gemfibrozil, and simfibrate. Aspirin is an antiplatelet drug. Drugs used for gout management include primarily xanthine oxidase inhibitors, such as allopurinol and febuxostat, and those used for uric acid management include primarily uricosuric agents, such as benzbromarone, probenecid, and sulfinpyrazone.

### 2.6. Exposure to Statins

The anatomical therapeutic chemical (ATC) classification system of the World Health Organization Collaborating Centre for Drug Statistics Methodology (WHOCC) was used to provide the ATC code [19] for the drugs included into the study’s analysis, and the registry for drug prescriptions (DRUG) file from the NHIRD was used to find the pharmacological composition and corresponding drug codes of the related drugs, thus obtaining outpatient and inpatient medication records of the study subjects. The ATC classification system classifies the active ingredients of drugs according to the organ or system on which they act and their therapeutic, pharmacological, and chemical properties, and sets the corresponding defined daily dose (DDD) for each drug. DDD is the standard unit for drugs recommended by the WHO to be used in research to internationally measure drugs. The definition is based on the assumed average daily maintaining dosage used by a 70-kg adult for the drug’s primary indication. Exposure of the drugs included in this study were measured and calculated using DDD as the unit, and the following equation was used to calculate the number of DDDs for each prescription of medical usage: Furthermore, in consideration of changes in exposure dosage during the long-term follow-up of personal drugs, cumulative DDDs (cDDDs) were calculated for the follow-up period of each study subject using prescription records of statins medication orders during the follow-up period for each study subject after the onset of chronic kidney disease or until the time of disease onset or the subject was lost to follow-up, and this was used as the drug exposure dose for each study subject.

### 2.7. Demographic Data, Including Comorbidity and Socio-Economic Status 

The residence of research subjects was inferred based on the insurance records of the insured and their medical records in the NHIRD. In addition, the degree of urbanization was based on the population density, education level, ratio of population age ≥65 years, development of industries, and distribution of medical resources, which categorized Taiwan into four clusters as follows: “highly urbanized cities and towns,” “emerging cities and towns,” “regular townships, cities, and districts,” and “agricultural and remote townships.”

In addition, patterns for smoking and alcohol use of the subjects were are not included in the NHIRD, although these two habits in daily life may seriously influence the results of this research. Therefore, this study used chronic obstructive pulmonary disease (COPD; ICD-9-CM: 491.x, 492.x) and alcohol-related diseases (ARD; ICD-9-CM: 291.x, 303.0, 303.9, 305.0, 571.0-571.3) to serve as the alternative variable indicators for chronic smoking and exposure to alcohol use, respectively [20].

### 2.8. Data Statistics and Analysis

#### 2.8.1. Descriptive Statistics

The study conducted descriptive statistical analysis according to statins usage by the study subjects on the incidence of sarcopenia, statins dosage used, related risk factors, comorbidity distribution, use of other related drugs, and demographic distribution including gender, age, income, and urbanization level. The Chi-square test was used to analyze differences between the categorical variables, and ANOVA was used to conduct univariate analysis in the differences of the continuous variables. These analyses were performed to infer if there was a difference between the usage of statin drugs regarding the factors mentioned above.

#### 2.8.2. Analytical Statistics

In the analysis of the relationship between statins drug usage and sarcopenia incidence in the chronic kidney disease population, the study subjects were categorized into groups according to the statins cDDDs during the follow-up period. Those with cDDDs <28 were defined as the non-exposure group, and the exposure groups were categorized into the three groups: low-exposure group, 28–89cDDDs; medium-exposure group, 90–180cDDDs; and high-exposure group, >180cDDDs. This study adopted the Cox proportional hazards model to conduct preliminary analysis on the effect of statins usage on the occurrence of newly diagnosed sarcopenia; further analysis was conducted using the Cox proportional hazards model with time-dependent covariates taking into consideration the individual temporal differences in medication usage, and calculated the hazard ratio (HR) and 95% confidence interval after controlling for gender, age, income, and urbanization.

## 3. Results

Table 1 shows that 37,215 (55.54%) patients were male. The mean follow-up time was 6.7 years, mean age of the patients was 60.5 years. Patients ≥65 years had the highest proportion of chronic kidney disease (44.65%). In terms of the economics aspect, those with zero monthly income (insured amount) accounted for the highest proportion (32.17%). For urbanization level of residence, those who resided in second level urbanization areas accounted for the highest proportion (45.61%). There were 2407 cases of newly diagnosed sarcopenia during the follow-up period in patients with chronic kidney disease. Among all subjects studied, 17,634 (26.3%) were using statins and 49,367 (73.7%) were not. Of those who were newly diagnosed with sarcopenia, 547 of them were using statins and 1860 of them were not. Mean follow-up time for those who were using statins and those who were not were 7.5 and 6.3 years, respectively. With respect to economics and urbanization level, those with zero monthly income (insured amount) and those who resided in second level urbanization areas accounted for the highest proportion in both the groups who were using statins and those who were not.

Table 2 shows the comorbidities in patients with chronic kidney disease. Of all the patients, 63.37% had hypertension, 46.80% hyperlipidemia, 10.01% atrial fibrillation and flutter, 21.33% heart failure, 40.36% coronary artery disease, 12.04% peripheral artery disease, 44.15% diabetes, 6.94% liver diseases, 23.10% chronic obstructive pulmonary diseases. All the comorbidities reached statistically significant difference between the group of patients with or without receiving statins.

The cDDDs levels during the follow-up period were analyzed for statins usage. It was found that 6.33%, 4.98%, and 15.01% of those in the low-, medium-, and high-exposure groups (2889, 90,180, and (>180 cDDDs) used statins, respectively. As for the usage of other drugs related to chronic kidney disease, 34.14% of patients used aspirin and 61.73% used NSAIDs. As for drugs other than statins for the management of hyperlipidemia, there were 2.77% and 12.5% of patients who used triglyceride lowering drugs and uricosuric agents, respectively. Among them, calcium channel blocker was the drug of choice for antihypertensive drugs and 50.06% patients used it, whereas benzbromarone was the uricosuric agent with the highest proportion (2.19%). Besides, 1.84% of patients used drugs for gout management.

Table 3 displays the risk assessment results for new onset sarcopenia in patients with chronic kidney disease who were using statins versus those who were not, as calculated by the Cox proportional hazard model. The study found the HR for the group that was using statins was 0.709 (95% CI, 0.645–0.780). After controlling for age, income, urbanization, comorbidities, and related drug usage, the adjusted HR of new onset sarcopenia for the group that was using statins was 0.753 (0.671–0.845). For analysis according to the statins cDDDs stratification, HR for new onset sarcopenia for the low-, medium-, and high-exposure groups was 0.909 (0.763–1.069), 1.063 (0.895–1.263), and 0.547 (0.480–0.622), respectively. In addition, adjusted HR for the low-, medium-, and high-exposure groups was 0.909 (0.770–1.069), 1.095 (0.912–1.313), and 0.577 (0.498–0.669), respectively. The study found that after adjusting for possible influencing factors, the medium-exposure group showed a higher HR, but this did not reach statistical significance, and the high-exposure group showed statistically significant protective effect.

Due to further consideration on the difference in time of drug usage between individuals, Cox proportional hazards model was employed with time-dependent covariates and controlling for age, gender, income, urbanization, comorbidities, and other related drug usage to calculate the risk for new onset sarcopenia in patients with chronic kidney disease that were using statins (Table 4). The adjusted HR was 0.963 (0.835–1.109), 1.241 (1.043–1.478), 0.771 (0.598–0.994), and 0.638 (0.453–0.899) for those using statins, those in the low-, medium-, and high-exposure groups, respectively. The results showed a significant dose-responsive trend.

**Figure 1:**
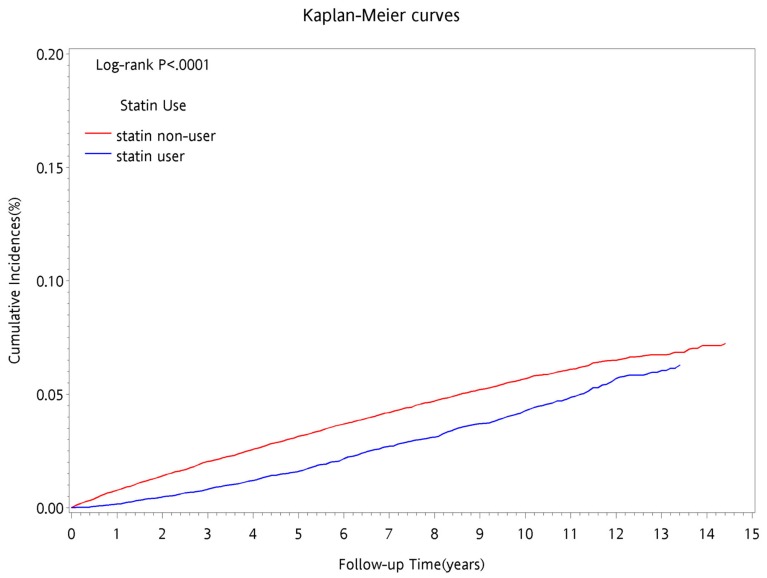
Cumulative incidence of sarcopenia by statins use among CKD patients in Taiwan during follow time.

The Kaplan–Meier method was used to estimate cumulative incidence of new onset sarcopenia during the follow-up period. The cumulative incidence of new onset sarcopenia during the follow-up period for the group that was using statins was lower than that for the group that was not using statins (Figure 1). Additionally, in terms of the follow-up period, the cumulative incidence rate of new onset sarcopenia for the group that was using statins gradually approached the cumulative incidence rate for the group that was not using statins, with significant difference (*p* < 0.0001) using the log-rank test.

Statins can be divided by their pharmacological characteristics into the two main categories as hydrophilic and lipophilic. The adjusted HR of new onset sarcopenia in patients with chronic kidney disease who were using hydrophilic statins in comparison to those who were not using statins was 1.013 (95% CI, 0.826–1.241; Table 5). The cDDDs stratification results showed that for those who were using hydrophilic statins, the adjusted HR showed decreased incidence risk for sarcopenia in the medium- and high-exposure groups, with a significant dose-responsive trend (*p* = 0.0283). The adjusted HR of new onset sarcopenia in patients with chronic kidney disease who were using lipophilic statins in comparison to those who were not using statins was 0.912 (95% CI, 0.763–1.090; Table 6). The cDDDs stratification results showed that, for those who were using lipophilic statins, the adjusted HR decreased the incidence risk for sarcopenia in the medium- and high-exposure groups, with a significant dose-responsive trend (*p* = 0.0066). Table 7 shows the risk assessment results comparing sarcopenia incidence for those who were using lipophilic and hydrophilic statins. The results showed that regardless of dosage stratification or correction for influencing factors, the risk for sarcopenia incidence was lower for those who were using lipophilic statins than for those who were using hydrophilic statins with no significant difference. Nonetheless, both lipophilic and hydrophilic statins displayed a protective trend.

Commonly used statins can be further subdivided into six primary categories with different therapeutic and harm effects due to slight differences in their pharmacodynamics effect, metabolic pathway, and pharmacological mechanism. Table 8 shows the comparison of risk assessment results of new onset sarcopenia for those who were using the six types of statins and for those who were not using statins. The results found that before adjustment, all types of statin drugs presented a decreased incidence risk for sarcopenia. After controlling for age, gender, income, urbanization, comorbidities, and related drug usage, the adjusted HR for fluvastatin was 0.883 (95% CI, 0.733–1.064) and for lovastatin was 0.924 (0.762–1.121). With the exception of the two drugs mentioned above which did not reach statistically significant differences, the remaining statin drugs all showed significantly decreased risk for incidence of sarcopenia. In addition, usage of any of the six types of statins all showed a decreasing trend for HR of sarcopenia incidence when compared to those who were not using statins.

Sensitivity analysis results for the effects of statin on newly onset sarcopenia among CKD patients in Taiwan with time-dependent covariates are shown in Table 9. Past literature has shown that gender, age, menopausal women, dialysis, and hyperlipidemia may be important risk factors for sarcopenia. The sensitivity analysis results showed that statistically significant differences were found in men (*p* = 0.0016) and in the age stratification group younger than 65. There was no significance difference of the risk of sarcopenia caused by the use of statins in women before and after menopause. There is a statistically significant difference in the risk of sarcopenia caused by the use of statins in patients with hyperlipidemia (*p* = 0.0007), which shows that the higher the dose, the more significant the protective effect. Because there were only 404 dialysis patients and there were only few patients with dialysis who were diagnosed sarcopenia with usage of statins, so they were not statistically analyzed. In addition, when the diagnosis department of sarcopenia was used in stratification, the results showed that the risk of sarcopenia caused by the use of statin drugs in non-home medicine diagnosis was statistically significant (*p* = 0.0011). However, whether or not it is diagnosed in a family medicine department, the risk of sarcopenia caused by the use of statins has a dose-response effect. The higher the dose, the better the protective effect.

## 4. Discussion

### 4.1. Investigation on Statins Usage and Sarcopenia

Previous research have pointed out that patients with chronic kidney disease often suffer from metabolic acidosis, which may lead to increase in protein catabolism, decrease in protein synthesis, insulin resistance, chronic inflammation, decrease in serum leptin concentration that may cause increase in the breakdown of branched amino acids and muscle proteins, thus inhibiting muscle protein synthesis [21,22,23,24] and resulting in increased occurrences of sarcopenia in late stage patients with chronic kidney disease [25]. However, there has been no research related to the exploration on the usage of statins in patients with chronic kidney disease and the incidence risk of sarcopenia. The results of this study showed that patients with chronic kidney disease who use statins can effectively decrease the incidence risk of sarcopenia, and the protective effect is most significant for those who use high dosage statins. Possible reasoning for this could be that chronic kidney patients may have higher risk for cardiovascular diseases, diabetes, and cerebral stroke. During the follow-up period of this study, usage of statins can prevent mortality from cardiovascular diseases in this particular population of patients and may even reduce the rate of Glomerular filtration rate GFR reduction thus decreasing kidney functional injury [26,27,28]. Furthermore, it could decrease the deterioration of proteinuria [26], thus delaying the starting time for dialysis treatment [29] and indirectly increasing muscle protein preservation. Therefore, there is a protective effect in statins usage for patients with chronic kidney disease regarding sarcopenia incidence risk. Furthermore, weight loss and changing life habits were also one of the important goals in addition to medication treatment in the 2015 Taiwan Chronic Kidney Disease Clinical Guidelines [30]. Therefore, change in life habits, increase in activity, and weight reduction may influence statins effects on the risk of newly onset sarcopenia.

### 4.2. Investigation on Related Factors of the Six Types of Statins and Sarcopenia

Sarcopenia is the combination of age-related progressive muscle bulk reduction and the decrease in muscle function and body performance [31]. Mikus et al. collected 37 patients between the age of 25–59 years who had metabolic syndromes and randomly assigned the patients to the two groups of aerobic exercise or aerobic exercise plus simvastatin administration (40 mg each day) [32]. The experiment time was 12 weeks and results showed that the group that underwent exercise with simvastatin administration had a 4.5% (*p* < 0.05) decrease in citrate synthase activity in skeletal muscles; this showed that although simvastatin can increase cardiopulmonary health and reduce abnormal blood lipid concentration, it can also cause skeletal muscle injury. Panza conducted three different studies on three different populations to evaluate how statins induce muscle power changes in patients with myopathy [33]. The first study randomly assigned atorvastatin (80 mg) or a placebo to healthy individuals and assessed the effects on muscle function; the second study administered simvastatin + placebo or simvastatin + coenzyme Q to patients who suffered from muscle soreness and evaluated the muscle power and symptoms before and after treatment; the third study provided different types of drugs, including rosuvastatin, simvastatin, pravastatin, in different dosages to clinical patients who were experiencing muscle pain. The results for the first study showed that subjects who used atorvastatin had a decrease in muscles, but the phenomenon was also observed in the placebo group, thus showing that the change in muscle power was not specific to the usage of atorvastatin. In the second study with the randomized cross-experiment using simvastatin and placebo, it was shown that the subjects who used simvastatin did not show any decrease in muscle power. For the third study, it was shown that different types of statins had different effects regarding risk for decrease in muscle power. For all three studies, the observed change in muscle power all showed no statistical significant, thus displayed that in the short term, the adverse effect of statins is unrelated to functional changes and different types of statins have different effects on muscle power and muscle function, displaying a possible difference in mechanism for the risk to cause sarcopenia.

This study performed stratified analysis in the six types of statins, and the results, after controlling for influencing factors, showed no statistical significant differences for fluvastatin and lovastatin. However, all six types of statins showed a protective effect regarding new onset sarcopenia. In addition, the study found that lipophilic statins had lower incidence risk for the onset of sarcopenia than hydrophilic statins, and this did not reach a statistical significant difference. Thus, this topic requires further exploration in the future.

## 5. Conclusions

Our main findings of this retrospective and large-scaled cohort study indicated that patients with chronic kidney disease who use statins seem to effectively prevent patients from occurrences of sarcopenia, high dosage of statins seem to show more significant protective effects, and the results are similar over long-term follow-up. In addition, the study observed that usage of hydrophilic statins has a higher risk for newly onset sarcopenia than the use of lipophilic statins. However, the study was limited to a Taiwanese population, the findings and conclusions thus may not be generalized to other populations.

Patients with chronic kidney disease are a susceptible population, and furthermore, a high risk population for sarcopenia, which could significantly affect chances for comorbidities such as cardiovascular diseases and increase mortality risk. Therefore, special protective measures should be provided for patients with chronic kidney disease to avoid comorbid harm from sarcopenia that may cause risks such as loss of muscle mass, decrease in muscle power, decrease in walking speed, and decrease in daily activity function. The results of this study showed that patients with chronic kidney disease given statins treatment showed effects regarding risk for newly onset sarcopenia. In the future, the usage of statins in patients with chronic kidney disease could be considered to decrease the incidence of newly diagnosed sarcopenia. 

### 5.1. Research Advantages

1. This study used the Longitudinal Health Insurance Database from the 2000 Registry for Beneficiaries of the Taiwan NHIRD, which is a large study sample size with good representativeness and low possibility for selection bias.
2. The NHIRD contains actual medical record of patients, thus decreasing recall bias.
3. The study subjects were selected based on disease diagnosis codes, and the disease diagnosis and coding were all processed by physicians, thus decreasing the occurrence of misclassification.
4. The follow-up time of this study is 15 years, and provides results and trends on the incidence risk for sarcopenia in patients with chronic kidney disease who use statins on the long-term.

### 5.2. Research Limitations

First of all, important influencing factors, such as laboratory data (serum and biochemistry examination), life habits (smoking and alcohol), family history (hereditary diseases), and surrounding living environment quality (exposure to pollution), could not be obtained from the NHIRD, and thus could not be corrected. Second, this study presumed that all study subjects took the prescribed statins medication according to medical order. However, there is still a possibility that some of the subjects may not have adhered to the medical orders and did not take the medication, thus the study had overestimated the actual statins dosage used by the study subjects. Third, the NHIRD did not include detailed information on laboratory data. For example, we were unable to estimate glomerular filtration rate or detect proteinuria at baseline and could not adjust for these unmeasured potential confounders. Additionally, actual examination values of muscle power, walking speed, and skeletal muscle mass of the newly onset sarcopenia subjects could not be known.

## Figures and Tables

**Table 1:** Demographic characteristics distribution regarding statins use of a chronic kidney disease population in Taiwan.

	All CKD		Statin User		Non-Statin User		
	(N = 67,001)		(N = 17,634)		(N = 49,367)		
Variable	No.	%	No.	%	No.	%	*p* Value
Sarcopenia							
Over all Sarcopenia	2407	3.59	547	3.10	1860	3.77	<0.0001
Dialysis	404	0.60	84	0.48	320	0.65	0.0114
Age of CKD onset ^†^							<0.0001
<45	12,777	19.07	2157	12.23	10,620	21.51	
45–65	24,308	36.28	8550	48.49	15,758	31.92	
>65	29,916	44.65	6927	39.28	22,989	46.57	
Med(IQR)	62.2 (48.70–73.50)		61.1 (51.70–70.00)		62.9 (47.30–74.90)		
Mean ± SD	60.50 ± 16.70		60.30 ± 13.00		60.50 ± 17.80		
Male sex	37,215	55.54	8839	50.12	28,376	57.48	<0.0001
Monthly income ^†^, NT$							<0.0001
0	21,533	32.17	6593	37.39	14,960	30.30	
1–15,840	14,152	21.12	3490	19.79	10,662	21.60	
15,841–25,000	20,756	30.98	4915	27.87	15,841	32.09	
≧25,000	10,540	15.73	2636	14.95	7904	16.01	
Urbanization level ^†^							<0.0001
I	16,209	24.19	4730	26.82	11,479	23.25	
II	30,560	45.61	8112	46.00	22,448	45.47	
III	13,447	20.07	3265	18.52	10,182	20.63	
IV	6785	10.13	1527	8.66	5258	10.65	
Follow time							
Med(IQR)	6.7(2.70-10.40)		7.5 (4.20–10.70)		6.30 (2.20-10.20)		
Mean ± SD	6.70 ± 4.30		7.40 ± 3.90		6.40 ± 4.40		

CKD, chronic kidney disease; NT$, New Taiwan dollars; Med (IQR), median (interquartile range); 95% CI, 95% confidence interval. Urbanization level: I indicates the highest level of urbanization and IV the lowest. ^†^ χ² test.

**Table 2:** Clinical characteristics of chronic kidney disease patients in Taiwan.

**Variable**	**All CKD**		**Statin User**		**Non-Statin User**		
	**(N = 67,001)**		**(N = 17,634)**		**(N = 49,367)**		
	**No.**	**%**	**No.**	**%**	**No.**	**%**	***p* Value**
Comorbidity							
Hypertension	42,456	63.37	15,067	85.44	27,386	55.48	<0.0001
Hyperlipidemia	31,354	46.80	15,488	87.83	15,866	32.14	<0.0001
Atrial fibrillation	6709	10.01	1933	10.96	4776	9.67	<0.0001
Heart failure	14,293	21.33	4808	27.27	9485	19.21	<0.0001
Coronary artery disease	27,044	40.36	10,018	56.81	17,026	34.49	<0.0001
Peripheral arterial disease	8070	12.04	3406	19.31	4664	9.45	<0.0001
Diabetes disease	29,584	44.15	12,261	67.53	17,323	35.09	<0.0001
Liver disease	4650	6.94	806	4.57	3844	7.79	<0.0001
COPD	15,477	23.10	4469	25.34	11,008	22.3	<0.0001
ARD	2831	4.23	553	3.14	1828	3.70	0.0005
Medical medicine†							
Statin							<0.0001
<28cDDDs	49,367	73.68	0	0.00	49,367	100.00	
28-89cDDDs	4239	6.33	4239	24.04	0	0.00	
90-180cDDDs	3336	4.98	3336	18.92	0	0.00	
>180cDDDs	10,059	15.01	10,059	57.04	0	0.00	
Statins type							
Pravastatin	2455	3.66	2455	13.92	0	0.00	
Fluvastatin	3438	5.13	3438	19.50	0	0.00	
Atorvastatin	9090	13.57	9090	51.55	0	0.00	
Lovastatin	2932	4.38	2932	16.63	0	0.00	
Simvastatin	5192	7.75	5192	29.44	0	0.00	
Rosuvastatin	5522	8.24	5522	31.31	0	0.00	
**Variable**	**All CKD**		**Statin User**		**Non-Statin User**		
	**(N = 67,001)**		**(N = 17,634)**		**(N = 49,367)**		
	**No.**	**%**	**No.**	**%**	**No.**	**%**	***p* Value**
Medical medicine†							
Aspirin	22,873	34.14	9957	56.46	12916	26.16	
NSAIDs	41,357	61.73	13,131	74.46	28226	57.18	
Antihypertensive drugs							
ACEi	21,536	32.14	8873	50.32	12663	25.65	<0.0001
ARB	24,839	37.07	11,391	64.60	13448	27.24	<0.0001
Beta Blocker	22,283	33.26	9608	54.49	12675	25.68	<0.0001
CCB	33,544	50.06	12,638	71.67	20906	42.35	<0.0001
Diuretic	29,650	44.25	10,624	60.25	19026	38.54	<0.0001
Anithyperglycemia drugs							
Insulins	14,324	21.38	5752	32.62	8572	17.36	<0.0001
Biguanides	16,429	24.52	8364	47.43	8065	16.34	<0.0001
Sulfonylureas	19,157	28.59	9148	51.88	10009	20.27	<0.0001
Thiazolidinedione	6210	9.27	4037	22.89	2173	4.40	<0.0001
Antihyperlipidemic drugs							
Nonstatin lipid-lowering drug	1854	2.77	1389	7.88	465	0.94	<0.0001
Fibrate	8373	12.50	5344	30.31	3029	6.14	<0.0001
Antihyperuric acid drugs							
Benzbromarone	1469	2.19	694	3.94	775	1.57	<0.0001
Probenecid	7	0.01	3	0.02	4	0.01	0.3893
Antihyperuric gout drugs							
Allopurinol	1235	1.84	598	3.39	637	1.29	<0.0001

Abbreviations: CKD, chronic kidney disease; COPD, chronic obstructive pulmonary disease; ARD, alcohol-related disease; ACEi, angiotensin-converting enzyme inhibitor; ARB, angiotensin Ⅱ receptor blocker; CCB, calcium channel blocker; NSAIDs, nonsteroidal anti-inflammatory drugs. cDDDs, cumulative defined daily doses.

**Table 3:** Association between statin and first-time sarcopenia among chronic kidney disease (CKD) patients in Taiwan.

Variable	Crude Sarcopenia HR	*p* Value	Adjust Sarcopenia HR	*p* Value
	(95%CI)		(95% CI)	
Statin Non-user(<28 cDDDs)	1		1	
Statin User(≥28 cDDDs) *	0.709 (0.645–0.780)	<0.0001	0.753 (0.671–0.845)	<0.0001
Statin use level *				
28–89cDDDs	0.909 (0.763–1.069)	0.2373	0.919 (0.770–1.096)	0.3453
90–180cDDDs	1.063 (0.895–1.263)	0.4843	1.095 (0.912–1.313)	0.3315
>180cDDDs	0.547 (0.480–0.622)	<0.0001	0.577 (0.498–0.669)	<0.0001

Abbreviations: HR, hazard ratio; 95% CI, 95% confidence interval; cDDDs, cumulative defined daily doses. * Statin use and categorization was defined by cumulative prescription (cDDDs) of statin. Models adjusted gender, age, income, urbanization, comorbidity.

**Table 4:** Association between statin and first-time sarcopenia among CKD patients in Taiwan with time- dependent covariates for statin.

Variable	Crude Sarcopenia HR	*p* Value	Adjust Sarcopenia HR	*p* Value
	(95% CI)		(95% CI)	
*Statin Non-User (<28 cDDDs)*	**1**		**1**	
Statin User (≥28 cDDDs) *	0.845 (0.741–0.965)	0.0129	0.963 (0.835–1.109)	0.5987
Statin use level *		<0.0001		0.0004
28–89cDDDs	1.113 (0.941–1.316)		1.241 (1.043–1.478)	
90–180cDDDs	0.675 (0.526–0.865)		0.771 (0.598–0.994)	
>180cDDDs	0.543 (0.387–0.762)		0.638 (0.453–0.899)	

Abbreviations: HR, hazard ratio; 95% CI, 95% confidence interval; cDDDs, cumulative defined daily doses.* Statin use and categorization was defined by cumulative prescription (cDDDs) of statin. Models adjusted gender, age, income, urbanization, comorbidity.

**Table 5:** Association between hydrophilic of statins and first-time sarcopenia among CKD patients in Taiwan.

Variable	Crude Sarcopenia HR (95% CI)	*p* Trend	Adjust Sarcopenia HR (95% CI)	*p* Value
Statin Non-user (<28 cDDDs)	**1**		**1**	
Hydrophilic Statin User	0.895 (0.735–1.090)	0.2706	1.013 (0.826–1.241)	0.9046
Hydrophilic Statin use level *		0.015		0.0283
28–89cDDDs	1.275 (0.960–1.692)		1.404 (1.053–1.870)	
90–180cDDDs	0.827 (0.583–1.720)		0.933 (0.655–1.327)	
>180cDDDs	0.589 (0.391–0.889)		0.684 (0.452–1.036)	

* Statin use and categorization was defined by cumulative prescription (cDDDs) of statin. Models adjusted gender, age, income, urbanization, comorbidity.

**Table 6:** Association between lipophilic of statins and first-time sarcopenia among CKD patients in Taiwan.

Variable		Crude Sarcopenia HR (95% CI)	*p* Trend	Adjust Sarcopenia HR (95% CI)	*p* Trend
Statin Non–user (<28 cDDDs)		**1**		**1**	
Lipophilic Statin User		0.812 (0.685–0.964)	0.0172	0.912 (0.763–1.090)	0.3121
Lipophilic Statin use level *			0.0009		0.0066
28–89cDDDs		1.046 (0.853–1.282)		1.157 (0.938–1.426)	
90–180cDDDs		0.571 (0.403–0.809)		0.646 (0.454–0.919)	
>180cDDDs		0.468 (0.259–0.846)		0.543 (0.299–0.984)	

Abbreviations: HR, hazard ratio; 95% CI, 95% confidence interval; cDDDs, cumulative defined daily doses. * Statin use and categorization was defined by cumulative prescription (cDDDs) of statin. Models adjusted gender, age, income, urbanization, comorbidity.

**Table 7:** Association between lipophilic and hydrophilic of statins and first-time sarcopenia among CKD patients in Taiwan.

Variable	Crude Sarcopenia HR (95% CI)	*p* Trend	Adjust Sarcopenia HR (95% CI)	*p* Trend
Hydrophilic User	**1**		**1**	
Lipophilic Statin User	0.919 (0.712–1.185)	0.5136	0.933 (0.723–1.205)	0.5961
Lipophilic Statin use level *				
28–89cDDDs	0.825 (0.585–1.165)	0.2752	0.856 (0.606–1.210)	0.3789
90–180cDDDs	0.685 (0.419–1.120)	0.1317	0.698 (0.426–1.144)	0.1542
>180cDDDs	0.842 (0.410–1.729)	0.6394	0.866 (0.419–1.786)	0.6961

Abbreviations: HR, hazard ratio; 95% CI, 95% confidence interval; cDDDs, cumulative defined daily doses. * Statin use and categorization was defined by cumulative prescription (cDDDs) of statin. Models adjusted gender, age, income, urbanization, comorbidity.

**Table 8:** Association between six types of statins and first-time sarcopenia among CKD patients in Taiwan.

Variable	Sarcopenia HR	*p* Value	Adjust Sarcopenia HR	*p* Value
	(95% CI)		(95% CI)	
**Statin Non-Use**	**1**		**1**	
Statin use	0.709 (0.645–0.780)	<0.0001	0.753 (0.671–0.845)	<0.0001
Hydrophilic Statin				
Pravastatin	0.681 (0.542–0.855)	0.0010	0.766 (0.607–0.968)	0.0257
Fluvastatin	0.785 (0.655–0.940)	0.0086	0.883 (0.733–1.064)	0.1917
Rosuvastatin	0.407 (0.330–0.501)	<0.0001	0.448 (0.362–0.555)	<0.0001
Lipophilic Statin				
Lovastatin	0.831 (0.690–1.001	0.0508	0.924 (0.762–1.121)	0.4238
Simvastatin	0.775 (0.667–0.902)	0.0010	0.850 (0.724–0.997)	0.0452
Atorvastatin	0.702 (0.620–0.795)	<0.0001	0.791 (0.691–0.906)	0.0007

Abbreviations: HR, hazard ratio; 95% CI, 95% confidence interval. Models adjusted gender, age, income, urbanization, comorbidity.

**Table 9:** Sensitivity analysis results.

	Statin Non–User	Statin Use Levels *						
		28–89 cDDDs		90–180 cDDDs		>180 cDDDs		
	HR	HR	95% CI	HR	95% CI	HR	95% CI	*p* Trend
**Main model ^†^**	1	1.241	1.043–1.475	0.771	0.598–0.994	0.638	0.433–0.899	0.0004
**Main model ^†^ plus:**								
**Medical diseases**								
** Hypertension**	1	1.192	1.001–1.420	0.737	0.572–0.950	0.609	0.432–0.858	0.0003
** Hyperlipidemia**	1	1.089	0.914–1.297	0.678	0.526–0.874	0.562	0.399–0.792	0.0001
** Atrial fibrillation**	1	1.24	1.042–1.477	0.77	0.597–0.993	0.637	0.452–0.898	0.0004
** Coronary heart disease**	1	1.212	1.018–1.443	0.745	0.587–0.960	0.605	0.430–0.853	0.0002
** Peripheral arterial disease**	1	1.231	1.034–1.466	0.76	0.590–0.980	0.625	0.444–0.881	0.0003
** Heart failure**	1	1.241	1.042–1.478	0.771	0.598–0.994	0.637	0.452–0.898	0.0004
**Diabeties disease**	1	1.196	1.004–1.424	0.743	0.577–0.959	0.612	0.434–0.862	0.0004
**Liver disease**	1	1.241	1.042–1.478	0.77	0.597–0.993	0.637	0.452–0.897	0.0004
** COPD**	1	1.247	1.047–1.485	0.774	0.600–0.997	0.638	0.453–0.899	0.0004
**Alcohol–related disease**	1	1.242	1.043–1.479	0.771	0.598–0.994	0.638	0.453–0.899	0.0004
** ** **Medication**								
** ** **Antihypertensive drugs**								
** ** **Diuretic**	1	1.222	1.026–1.455	0.754	0.585–0.972	0.62	0.440–0.874	0.0003
** ** **Beta Blocker**	1	1.238	1.040–1.475	0.768	0.596–0.991	0.635	0.450–0.895	0.0004
**Anithyperglycemia drugs**								
**Insulins**	1	1.229	1.032–1.464	0.755	0.585–0.973	0.613	0.435–0.864	0.0002
**Biguanides**	1	1.211	1.016–1.442	0.749	0.581–0.967	0.623	0.442–0.878	0.0004
**Sulfonylureas**	1	1.168	0.980–1.392	0.72	0.558–0.928	0.6	0.426–0.846	0.0003
**Thiazolidinedione**	1	1.218	1.022–1.451	0.75	0.581–0.968	0.616	0.437–0.869	0.0003
**Antihyperlipidemic drugs**								
**Nonstatin lipid–lowering drug**	1	1.242	1.043–1.478	0.771	0.598–0.994	0.639	0.453–0.9	0.0004
**Fibrate**	1	1.243	1.044–1.480	0.77	0.598–0.993	0.636	0.451–0.896	0.0004
**Aspirin**	1	1.152	0.967–1.373	0.703	0.545–0.907	0.57	0.404–0.804	0.0001
**NSAIDs**	1	1.212	1.018–1.443	0.755	0.586–0.973	0.624	0.443–0.879	0.0004
**Benzbromarone**	1	1.242	1.043–1.478	0.771	0.598–0.994	0.638	0.453–0.899	0.0004
**Antihyperuric gout drugs**								
**Allopurinol**	1	1.24	1.041–1.476	0.77	0.597–0.992	0.637	0.452–0.898	0.0004
**Subgroup analysis†**								
**Gender**								
**Female**	1	1.163	0.917–1.476	0.88	0.641–1.208	0.699	0.451–1.084	0.1596
**Male**	1	1.364	1.055–1.762	0.63	0.411–0.968	0.568	0.327–0.986	0.0016
**Age**								
**<65**	1	1.151	0.911–1.453	0.589	0.416–0.861	0.586	0.374–0.917	0.0017
**≥65**	1	1.329	1.023–1.727	1.003	0.703–1.430	0.667	0.391–1.136	0.0657
**Menopause**								
**Yes**	1	1.226	0.951–1.582	0.903	0.641–1.272	0.649	0.393–1.072	0.1004
**No**	1	0.744	0.375–1.478	0.698	0.305–1.600	0.85	0.343–2.109	0.7089
** Hyperlipidemia**								
**Yes**	1	1.191	0.984–1.441	0.743	6.566–6.976	0.583	0.402–0.846	0.0007
**No**	1	1.604	1.052–2.446	0.917	0.454–1.849	1.003	0.414–2.428	0.1776
**Family medicine**								
**Yes**	1	1.576	0.956–2.600	1.088	0.586–2.021	0.435	0.136–1.396	0.132
**No**	1	1.224	1.014–1.496	0.716	0.541–0.948	0.604	0.463–0.951	0.0011

Abbreviations: cDDDs, cumulative defined daily doses; HR, hazard ratio; 95% CI, 95% confidence interval; COPD, chronic obstructive pulmonary disease; ACEi, angiotensin–converting enzyme inhibitor; ARB, Angiotensin Ⅱ Receptor Blocker; Alpha–Gi, alpha–glucosidase inhibitor; NSAIDs, nonsteroidal anti-inflammatory drugs. * Statin use and categorization was defined by cumulative prescription (cDDDs) of statin. ^†^ Models adjusted gender, age, income, urbanization, comorbidity.
